# Supplementary material for: Polymorphisms in Processing and Antigen Presentation-Related Genes and Their Association with Host Susceptibility to Influenza A/H1N1 2009 Pandemic in a Mexican Mestizo Population
Source: Viruses. 2020 Oct 29;12(11):1224. doi: 10.3390/v12111224 (PMC7692058; doi:10.3390/v12111224)
Supplement: Supplementary file 1 [file viruses-12-01224-s001.pdf]

Polymorphisms in processing and antigen presentation-related genes and their association with host susceptibility to 2009 pandemic influenza A (H1N1) in a Mexican mestizo population

Marco Antonio Ponce-Gallegos[1], Aseneth Ruiz-Celis[1], Enrique Ambrocio-Ortiz[1], Gloria Pérez-Rubio[1], Alejandra Ramírez-Venegas[2], Nora E. Bautista-Félix, Ramcés Falfán-Valencia[1*]

Supplementary material

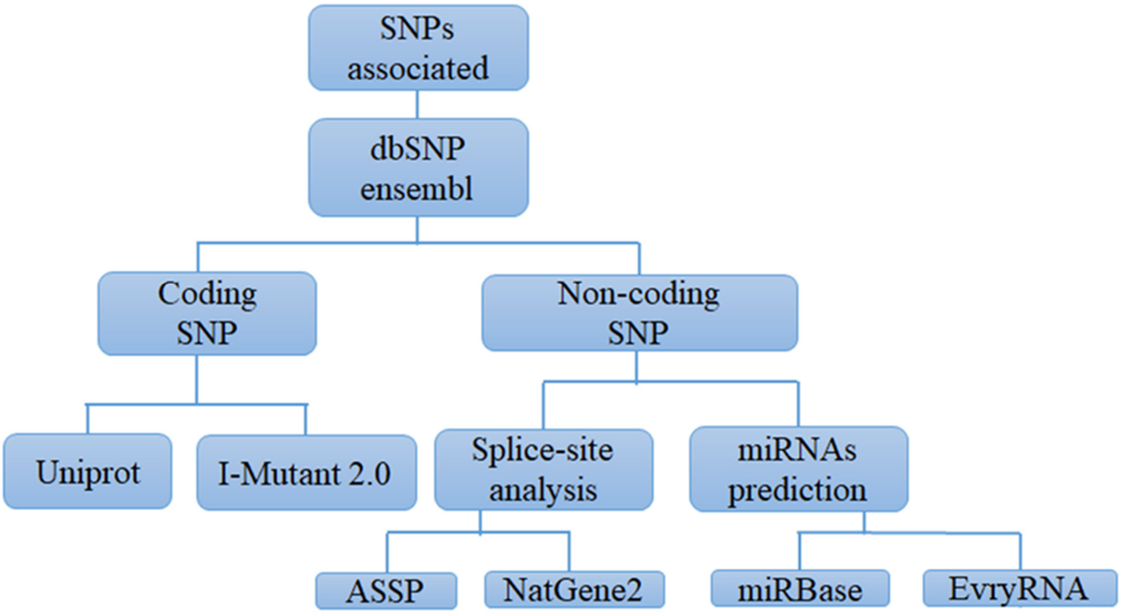

Supplementary Figure S1. Algorithm used for *in silico* analysis.

Supplementary Table S1. Molecular characteristics of the evaluated SNPs.

Genes	SNP	Position	Change		Consequence	MAF*	
			Nucleotide	Amino acid		Global	MXL
TAP1	rs1057149	Exon	C/T	R (Arg) > Q (Gln)	Missense variant	0.06	0.05
	rs2127679	Exon	G/A	A (Ala) > V (Val)	Missense variant	0.03	0.03
	rs4148882	Intron	A/G	N/A	Intron variant	0.26	0.25
	rs41561219	Exon	C/T	V (Val) > I (Ile)	Missense variant	0.03	0.03
TAP2	rs13501	Intron	G/A	N/A	Intron variant	0.4	0.42
	rs241433	Intron	A/C	N/A	Intron variant	0.46	0.44
	rs241441	Exon	T/C	G (Gly) > G (Gly)	Synonymous variant	0.3	0.29
	rs2071544	Intron	C/T	N/A	Intron variant	0.49	0.42
TAPBP	rs2071888	Exon	C/G	T (Thr) > R (Arg)	Missense variant	0.41	0.4
	rs2282851	Intron	C/T	N/A	Intron variant	0.22	0.38
PSMB8	rs2071542	Exon	A/G	A (Ala) > A (Ala)	Synonymous variant	0.05	0.11
	rs2071543	Exon	G/T	Q (Gln) > E (Glu)	Missense variant	0.15	0.19
	rs3763365	2KB Upstream variant	G/A	N/A	Upstream transcript variant	0.49	0.4
	rs9276810	Intron	G/A	N/A	Intron variant	0.44	0.49
PSMB9	rs17587	Exon	G/A/T	R (Arg) > H (His)	Missense variant	0.22	0.26
	rs241418	3'-UTR variant	G/A	N/A	3'-UTR variant	0.02	0.01
	rs2071534	Intron	C/T	N/A	Intron variant	0.44	0.51

SNP= Single nucleotide polymorphism; MAF= Minor allele frequency; MXL= Mexicans from Los Angeles. *Frequencies were obtained from the 1000 genomes project [23]

Supplementary Table S2. Non-comparable clinical features of INF-P group.

Variables	INF-P = 128
**Comorbidities**	
Hypertension (%)	17 (13.28)
Asthma (%)	10 (7.81)
Diabetes (%)	10 (7.81)
COPD (%)	4 (3.12)
**Symptomatology**	
Fever (%)	97 (75.78)
Dyspnea (%)	93 (72.66)
Cough (%)	68 (53.13)
Rhinorrhea (%)	42 (32.81)
Nasal congestion (%)	15 (11.72)
**Clinical variables**	
Leykocytes	7.45 (1.9-17.6)
Platelets	212 (21-551)
Glucose	108.3 (43-341)
BUN	12 (2-83)
Urea	27 (5-178)
Creatinine	0.82 (0.31-10.1)
CPK	259 (11-7241)
LDH	536 (104-2005)
AST	47 (35-252)
ALT	38 (17-174)
**Complications**	
Pneumonia (%)	76 (59.38)
ICU (%)	46 (35.94)
ARDS (%)	38 (29.69)
Death (%)	19 (14.84)

INF-P: Patients with Influenza A/H1N1 infection; COPD: Chronic obstructive pulmonary disease; BUN: blood urea nitrogen; CPK: Creatine phosphokinase; LDH: Lactic dehydrogenase; AST: Aspartate aminotransferase; ALT: Alanine aminotransferase; ICU: Intensive care unit admission; ARDS: Acute respiratory distress syndrome.

Supplementary Table S3. Hardy-Weinberg Equilibrium analysis.

Gene	SNP	HC		p-value
		Obs. Het	Pred. Het	
TAP1	rs1057149	0.161	0.148	0.396
	rs2127679	0.013	0.013	1
	rs4148882	0.464	0.433	0.364
	rs41561219	0.034	0.033	1
TAP2	rs13501	0.815	0.483	1.53E-30
	rs241433	0.571	0.408	1.82E-12
	rs241441	0.596	0.482	5.00E-04
	rs2071544	0.645	0.489	3.70E-06
TAPBP	rs2071888	0.636	0.497	3.31E-05
	rs2282851	0.62	0.499	3.00E-04
PSMB8	rs2071542	0.247	0.229	0.387
	rs2071543	0.378	0.36	0.575
	rs3763365	0.528	0.494	0.367
	rs9276810	0.525	0.497	0.485
PSMB9	rs17587	0.35	0.345	1
	rs241418	0.03	0.029	1
	rs2071534	0.494	0.495	1

Data of the 17 SNPs included in the study, as well as the heterozygote proportions. We present p-values for HWE in the control group. SNP: Single nucleotide polymorphism; HC: Healthy contacts; Obs. Het: observed heterogeneity; Pred. Het: predicted heterogeneity.

Supplementary Table S4. Allele and genotype frequencies from Case and Control groups of the non-significative SNPs evaluated.

Gene	INF-P		HC	
	n	F (%)	n	F (%)
TAP1	**rs1057149**			
**Genotypes**				
CC	108	85.04	91	82.73
CT	19	14.96	19	17.27
TT	0	0	0	0
	127	100	110	100
**Alleles**				
C	235	92.52	201	91.36
T	19	7.48	19	8.64
TAP1	**rs2127679**			
**Genotypes**				
GG	125	98.43	110	99.10
GA	2	1.57	1	0.90
AA	0	0	0	0
	127	100	111	100
**Alleles**				
G	250	99	220	99.55
A	2	1	1	0.45
TAP1	**rs4148882**			
**Genotypes**				
AA	63	49.61	43	39.45
AG	56	44.09	53	48.62
GG	8	6.30	13	11.93
	127	100	109	100
**Alleles**				
A	182	72	99	55.62
G	72	28	79	44.38
TAP1	**rs41561219**			
**Genotypes**				
CC	124	97.64	105	95.45
CT	3	2.36	5	4.55
TT	0	0	0	0
	127	100	110	100
**Alleles**				
C	251	99	215	97.73

T	3	1	5	2.27
TAP2		**rs13501**		
**Genotypes**				
GG	22	18.03	20	19.05
GA	100	81.97	85	80.95
AA	0	0	0	0
	122	100	105	100
**Alleles**				
G	144	59.02	125	59.52
A	100	40.98	85	40.48
TAP2		**rs241441**		
**Genotypes**				
TT	41	32.28	29	26.85
TC	73	57.48	67	62.04
CC	13	10.24	12	11.11
	127	100	108	100
**Alleles**				
T	155	61	125	57.87
C	99	39	91	42.13
TAP2		**rs2071544**		
**Genotypes**				
CC	29	24.58	26	25.49
CT	80	67.80	62	60.78
TT	9	7.63	14	13.73
	118	100	102	100
**Alleles**				
C	138	58	114	55.88
T	98	42	90	44.12
PSMB8		**rs2071542**		
**Genotypes**				
AA	89	70.08	86	79.63
AG	36	28.35	22	20.37
GG	2	1.57	0	0
	127	100	108	100
**Alleles**				
A	214	84.25	194	89.81
G	40	15.75	22	10.19
PSMB8		**rs2071543**		
**Genotypes**				

GG	75	58.59	62	56.36
GT	48	37.50	42	38.18
TT	5	3.91	6	5.45
	128	100	110	100
**Alleles**				
G	198	77	166	75.45
T	58	23	54	24.55
PSMB8	**rs3763365**			
**Genotypes**				
GG	39	31.45	28	27.72
GA	68	54.84	49	48.51
AA	17	13.71	24	23.76
	124	100	101	100
**Alleles**				
G	146	58.87	105	51.98
A	102	41.13	97	48.02
PSMB8	**rs9276810**			
**Genotypes**				
GG	33	25.78	32	29.09
GA	70	54.69	55	50.00
AA	25	19.53	23	20.91
	128	100	110	100
**Alleles**				
G	136	53.13	119	54.09
A	120	46.88	101	45.91
PSMB9	**rs17587**			
**Genotypes**				
GG	73	57.48	70	63.64
GA	47	37.01	36	32.73
AA	7	5.51	4	3.64
	127	100	110	100
**Alleles**				
G	193	75.98	176	80
A	61	24.02	44	20
PSMB9	**rs241418**			
**Genotypes**				
GG	122	96.83	108	97.30
GA	4	3.17	3	2.70
AA	0	0	0	0

	126	100	111	100
**Alleles**				
G	248	98	219	98.65
A	4	2	3	1.35
PSMB9		**rs2071534**		
**Genotypes**				
CC	34	27.20	37	33.64
CT	62	49.60	54	49.09
TT	29	23.20	19	17.27
	125	100	110	100
**Alleles**				
C	130	52	128	58.18
T	120	48	92	41.82

INF-P: Patients with influenza A/H1N1 infection; HC: Healthy contacts.
